# Identification and Characterization of mRNA Biomarkers for Sodium Cyanide Exposure

**DOI:** 10.3390/toxics9110288

**Published:** 2021-11-02

**Authors:** Min Kim, Seung-Cheol Jee, Soee Kim, Kyung-Hwa Hwang, Jung-Suk Sung

**Affiliations:** 1 Department of Life Science, Biomedi Campus, Dongguk University-Seoul, 32 Dongguk-ro, Ilsandong-gu, Goyang 10326, Gyeonggi-do, Korea; pipikimmin@naver.com (M.K.); markjee@naver.com (S.-C.J.); soeesoee@naver.com (S.K.); 2 Jeonbuk Branch, Korea Institute of Toxicology, KIT, KRICT, 30 Baehak 1-gil, Jeongeup-si 56212, Jeollabuk-do, Korea; khwang@kitox.re.kr

**Keywords:** biomarker, sodium cyanide, exposure assessment, RNA-seq

## Abstract

Biomarkers in exposure assessment are defined as the quantifiable targets that indicate the exposure to hazardous chemicals and their resulting health effect. In this study, we aimed to identify, validate, and characterize the mRNA biomarker that can detect the exposure of sodium cyanide. To identify reliable biomarkers for sodium cyanide exposure, critical criteria were defined for candidate selection: (1) the expression level of mRNA significantly changes in response to sodium thiocyanate treatment in transcriptomics results (fold change > 2.0 or <0.50, adjusted *p*-value < 0.05); and (2) the mRNA level is significantly modulated by sodium cyanide exposure in both normal human lung cells and rat lung tissue. We identified the following mRNA biomarker candidates: *ADCY5*, *ANGPTL4*, *CCNG2*, *CD9*, *COL1A2*, *DACT3*, *GGCX*, *GRB14*, *H1F0*, *HSPA1A*, *MAF*, *MAT2A*, *PPP1R10*, and *PPP4C.* The expression levels of these candidates were commonly downregulated by sodium cyanide exposure both in vitro and in vivo. We functionally characterized the biomarkers and established the impact of sodium cyanide on transcriptomic profiles using in silico approaches. Our results suggest that the biomarkers may contribute to the regulation and degradation of the extracellular matrix, leading to a negative effect on surrounding lung cells.

## 1. Introduction

Hazardous substances, whether artificially made or naturally occurring, can enter the human body through the main routes of exposure such as inhalation, ingestion, and dermal contact, and cause various effects on health [1]. Exposure to chemicals causes a wide range of health risks, such as irritation, sensitization, and carcinogenicity [2]. Controlling the exposure to hazardous chemicals and toxic substances is a fundamental way to protect workers. However, once exposed to hazardous substances, it is also important to measure the degree of exposure [3,4].

Human biomonitoring uses biomarkers to assess specific exposures and to predict the risk of adverse health effects in individuals and populations [5,6]. Biomarkers in exposure assessment refer to actual chemicals, chemical metabolites, or changes in organic matter in the body that indicates the exposure of an organism to a chemical [7]. Three types of biomarkers are used when dealing with exposure assessments; biomarkers of susceptibility, biomarkers of exposure, and biomarkers of effect [8,9,10]. They can identify not only whether the exposure has occurred, but also the route, toxic pathway, and effects of the exposure. These indicators also allow researchers to work in real-time for the determination of exposure and prevent further damage [11]. Biomarkers of exposure assessment are useful for further research on the chemical of interest, and the results may contribute to standards and guidelines regarding chemical exposure [12]. To use biomarkers as a predictor of exposure, it needs high specificity and strong correlation with a health effect. The direct method for assessing exposure to chemicals is to analyze the actual chemicals or their metabolites in blood and urine specimens [13]. However, this method has limitations in its application to chemicals that are rapidly metabolized and eliminated from the body [14]. Except for heavy metals, most of the chemicals are metabolized and excreted from the human body within 24 h [15]. Therefore, when a chemical incident occurs, it is difficult to measure exposure and risk assessment a few days after the chemical incident.

It is essential to discover and validate biomarkers applicable to children as well as adults [16]. Children are much more sensitive than adults to hazardous chemicals in the environment [17,18]. Children absorb more toxic chemicals per body weight than adults because children have a higher respiratory ventilation rate than that of adults [19,20]. Additionally, the metabolic pathways of children are immature, which have insufficient enzymes needed to metabolize and remove toxic chemicals from the body [21]. In addition, children’s organs such as the central nervous, immune, reproductive, and digestive systems are still developing, making them particularly vulnerable to toxic chemicals as the rate of cell division increases [22]. Therefore, biomarkers should be developed considering developmental properties in children.

Sodium cyanide, a highly poisonous inorganic compound, is widely used as an important precursor for many organic and inorganic chemicals in chemical manufacturing, including pharmaceuticals [23]. Smoke inhalation by household fire smoke and cigarette smoking is also common source of cyanide poisoning [24,25]. Cyanide is also released from bacteria such as *P. aeruginosa* through the metabolic pathway of bacterial cyanogenesis, leading to chronic lung disease and respiratory failure [26]. Sodium cyanide releases hydrogen cyanide gas, a highly toxic chemical that interferes with the body’s ability to use oxygen [27]. Acute exposure to sodium cyanide can be rapidly fatal, and sustained exposure to low concentrations affects organs that are sensitive to hypoxic levels such as the central nervous system, the cardiovascular system, and lungs [28]. The half-life of cyanide ions in the body is about 2 h, and thiocyanate ions, one of the metabolites of cyanide, is eliminated from the body in approximately 6 days [29,30].

In this study, we analyzed global mRNA expression modulation by sodium cyanide exposure. We identified mRNA biomarkers and validated their expression in vitro and in vivo. We functionally characterized the biomarkers and established the impact of sodium cyanide on transcriptomic profiles using in silico approaches.

## 2. Materials and Methods

### 2.1. Test Animals

A total of each 20 Sprague-Dawley (SD) rats (5 weeks of age) initially weighing approximately 120 ± 10 g were obtained from Yu-Han Co. (Anyang, Korea). They were housed at a density of five test animals per cage in a specific pathogen-free (SPF) room with a 12 h–12 h light–dark cycle and provided food and water ad libitum. The ambient air temperature and relative humidity were set to 23 ± 2 °C and 55 ± 4%, respectively. For the study using sodium cyanide, the test animals were randomly divided into four groups: (1) control group treated with water; and those treated with (2) 20 mg/kg, (3) 40 mg/kg, or (4) 80 mg/kg of sodium cyanide. The sodium cyanide (SIGMA-Aldrich, 380970, St. Louis, MO, USA) was dissolved in water and administered orally to the test animals by syringe. All animal protocols were prepared in accordance with the Animal Protection Act of Korea and Guide for the Care and Use of Laboratory Animals published by the Institute for Laboratory Animal Research (ILAR). These animal experiments were approved by the Institutional Animal Care and Use Committee (IACUC) of the Korea Institute of Toxicology (KIT).

### 2.2. Cell Culture and Treatments

The BEAS-2B cell line was purchased from the American Type Culture Collection (Manassas, VA, USA). The cells were cultured in the base medium (BEBM) along with all the additives (Lonza, Basel, Switzerland). All the cells were incubated at 37 °C in 5% CO₂, and the medium was changed once every 3–4 days. For the experiments, cells were seeded at 2 × 10^4^ cells/cm^2^ of density. To select the experimental concentration, various concentrations of sodium thiocyanate (0.25–400 μM) were administered for 4 h or 1 d. After the cell viability assay, the three-point concentrations of sodium thiocyanide were selected and administered to cells in subsequent experiments.

### 2.3. Cell-Viability Analysis

Cell viability was assessed using EZ-CYTOX reagent (DOGEN, Seoul, Korea) according to the manufacturer’s protocol. The cells were seeded into 96-well plates at 1 × 10^4^ cells/well, after treatment of sodium thiocyanate (SIGMA-Aldrich, 251410, St. Louis, MO, USA). For 4 h and 24 h, the cell culture medium containing the compounds was changed to the medium containing EZ-CYTOX reagent. After incubation for 2 h, the absorbance was measured under 450 nm using a microplate reader (Molecular Devices, San Jose, CA, USA). The cell viability of the sodium thiocyanate-treated group was compared with that of the vehicle-treated control group.

### 2.4. mRNA Extraction

Total RNA was extracted using TRIzol reagent (Life Technologies, Carlsbad, CA, USA), following the manufacturer’s protocol. RNA was dissolved in DEPC-treated H_2_O and used for Quantification Sequencing of 3′ mRNA and real-time RT PCR. To verify the RNA integrity, agarose-gel electrophoresis was performed using 1% agarose (Sigma-Aldrich, St. Louis, MO, USA) in TAE (Biosesang, Gyeonggi, Korea) and ETBR (Sigma-Aldrich, St. Louis, MO, USA). RNA was denatured in RNA gel loading dye containing formamide and run for 40 min with 50 V. To confirm the high RNA quality of all the samples, the RNA integrity number was checked to be >7.0. The quantity was determined by Nanodrop-2000 (Thermo Fisher Scientific, Waltham, MA, USA) with a ratio of absorbance of >1.8 at 260 and 280 nm.

### 2.5. mRNA Quantification Sequencing (Quan-Seq)

The RNA-sequencing assay was performed by E-Biogen Inc. (Seoul, Korea). Briefly, 2 μg of RNA was prepared and incubated with magnetic beads conjugated to oligo-dT, and RNAs other than mRNA were washed out. The library was randomly hybridized with Illumina-compatible linker sequences to the poly (A) RNA. The newly synthesized complementary DNA (cDNA) insert was ligated to the stopper. Second strand synthesis was performed to release the library from the beads, and then the library was amplified. For each RNA sample, a library was constructed using a QuantSeq 3′ mRNA-Seq Library Prep Kit (Lexogen, Vienna, Austria). Data mining and graphic visualization were performed using ExDEGA (E-Biogen, Inc., Seoul, Korea).

### 2.6. Gene Ontology (GO) and Pathway Analysis

GO and pathway analysis of differentially expressed genes (DEGs) was also performed. The analysis was performed using the genes whose expression levels were changed by ≥2 folds compared with the control levels, and a log_2_-normalized read count of target genes was applied greater than 10 to minimize false counts. Genes were classified based on DAVID (https://david.ncifcrf.gov/, accessed on 30 October 2021) and Medline databases (http://www.ncbi.nlm.nih.gov/, accessed on 30 October 2021).

### 2.7. Protein–Protein Interaction (PPI) Analysis

To analyze a functional interaction network, PPI mapping of DEGs was performed based on the RNA results. The interactions of each protein were predicted using the human interactome in STRING database version 11.0 (http://string-db.org/, accessed on 30 October 2021). The STRING database is based on a combination of various sources, such as experimental verification, text mining, and co-expression.

### 2.8. Real-Time Quantitative Reverse Transcription-Polymerase Chain Reaction (Real-Time qRT-PCR)

cDNA was synthesized from 2 μg of total RNA by using M-MLV (Moloney Murine Leukemia Virus) Reverse Transcriptase (ELPIS-BIOTECH, Daejeon, Korea) and used for qRT-PCR (CFX Connect™ Real-Time PCR Detection System; Bio-Rad, Hercules, CA, USA) with the SYBR Green PCR Master Mix (KAPA, Wilmington, MA, USA). Amplification was performed using the following cycling program: initial denaturation at 95 °C for 3 min, 40 cycles of denaturation at 95 °C for 10 s, annealing at 60 °C for 10 s, and extension at 72 °C for 10 s. To assess the real-time PCR reactions for primer-dimer artifacts and to ensure the specificity of the primers, post-amplification melting-curve analysis was performed.

### 2.9. Statistical Analysis

The data were analyzed using GraphPad Prism version 5.0 (GraphPad Software, Inc., San Diego, CA, USA) and are expressed as the mean ± SEM of three independent experiments. Statistical significance was analyzed using one-way ANOVA with Tukey’s multiple comparison analysis. A *p*-value < 0.05 was considered to indicate statistical significance.

## 3. Results

### 3.1. Identification of Biomarkers Associated with Sodium Cyanide Exposure

**Figure 1:**
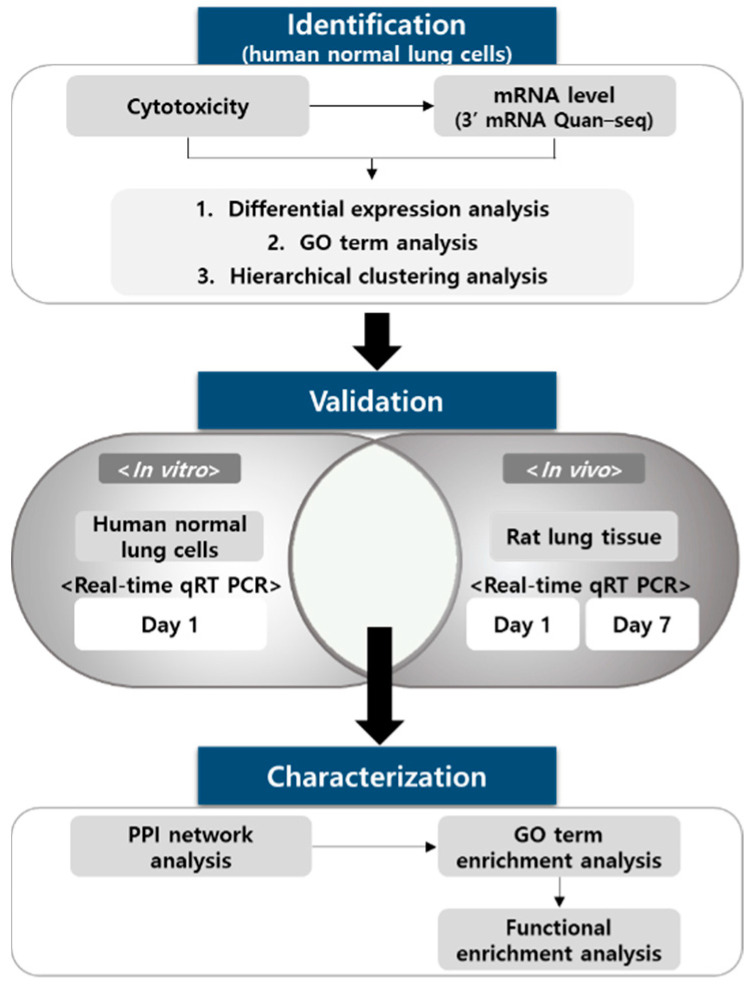
Workflow of the identification and characterization of biomarkers of sodium cyanide exposure. The DEGs after sodium thiocyanate exposure were analyzed via transcriptomic analyses. Then, the candidate mRNA biomarkers were validated at various treatment doses and time points in vitro and in vivo. For biomarker characterization, the network analysis of biomarkers, GO term, and functional enrichment analysis were performed. DEG, differentially expressed gene; GO, gene ontology.

**Figure 2:**
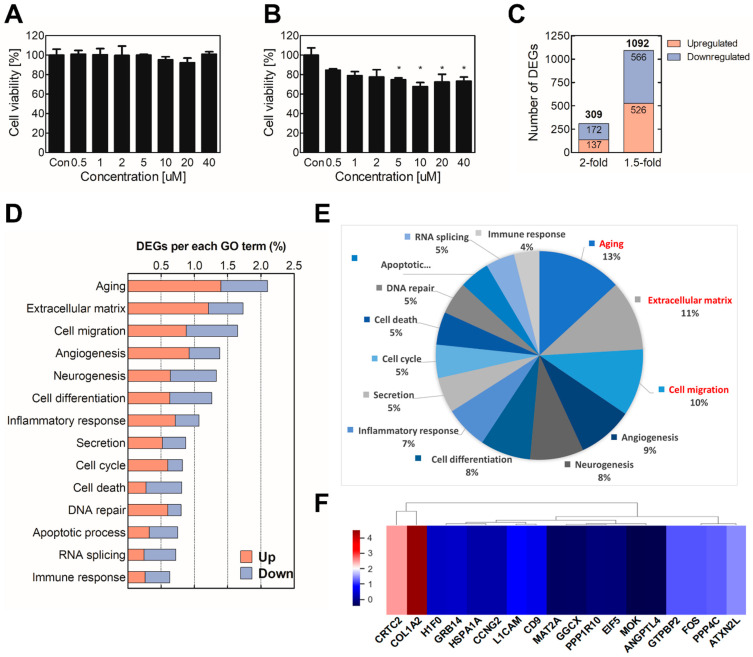
Identification of biomarker candidates for assessment of sodium cyanide exposure. To determine the cytotoxicity of sodium thiocyanate, BEAS-2B cells were treated with various concentrations of sodium thiocyanate for (**A**) 4 h or (**B**) 24 h. Cell viability was analyzed using the WST assay. Then, mRNA quan-seq was conducted using the total RNA of the cells treated with sodium thiocyanate. (**C**) The differential expression analysis was performed by comparing the mRNA levels of the sodium-thiocyanate–treated cells with those of the vehicle-treated control cells. (**D**) GO analysis was conducted using the DEGs (≥2 folds). (**E**) The proportions of the GO terms are presented in a circular diagram. (**F**) The heatmap of hierarchical clustering of the biomarker candidates was constructed using the z-scores of the genes. Red and blue indicate upregulation and downregulation, respectively. Each result is the mean ± SEM of at least three independent experiments. * *p* < 0.05. GO, gene ontology; DEG, differentially expressed gene.

The overall workflow is depicted in Figure 1. Sodium cyanide is rapidly metabolized and has a half-life of <24 h in vivo. The toxicity of sodium cyanide results from the metabolites. The major metabolite of sodium cyanide is sodium thiocyanate. Therefore, the BEAS-2B normal lung cell line was treated with sodium thiocyanate at various concentrations for 4 h and 1 d. Sodium thiocyanate showed no cytotoxicity in 4 h but had significant toxicity by 24 h in a dose-dependent manner (Figure 2A,B). The concentrations of 1, 10, and 20 μM of sodium thiocyanate were selected for the subsequent experiments.

To select biomarker candidates, the sodium thiocyanate was administered to BEAS-2B cells for 4 h, and then total mRNA was extracted and used for mRNA Quan-seq. Among 25,737 mRNAs, 1092 genes were differentially expressed by at least 1.5 folds (FC ≥ 1.5 or <0.67, normalized (log2) > 4, adjusted *p*-value < 0.05). Especially, the expression levels of 309 genes were significantly altered (≥2 folds) after sodium thiocyanate treatment (Figure 2C). Additionally, the DEG results showed that the gene expression levels were mostly downregulated rather than upregulated, suggesting that sodium thiocyanate tends to attenuate rather than induce the gene expression levels. The 309 significant DEGs were analyzed to distinguish the groups based on the biological processes in which they participated. The analysis was performed using the online bioinformatics analysis tool DAVID and a Gene Ontology (GO) Database that attaches hierarchical descriptors to all the annotated proteins encoded by the human genome. The Biological Process GO terms were applied to identify which Biological Process terms are represented statistically more frequently in a list of DEGs. The identified DEGs were mostly related to the extracellular matrix, aging, and cell migration (Figure 2D,E).

To analyze the similarity of DEGs and cluster individual biomarkers, 309 significantly modulated DEGs were standardized to the z-score and used for hierarchical clustering. To calculate the z-score, each biomarker was normalized using a z-transformation. The clustering heatmap provides an interactive visualization for the classification of DEGs by sodium thiocyanate exposure. Among them, the significant genes were extracted (Figure 2F). Based on the GO results and significant DEGs, 14 biomarker candidates were selected (Table 1).

### 3.2. Validation of the Candidate Biomarkers of Sodium Cyanide Exposure

To validate the applicability of biomarker candidates for exposure assessment of sodium cyanide, gene expression levels were analyzed in vitro and in vivo. For the in vitro experiments, 1, 10, or 20 μM of sodium thiocyanate were administered to BEAS-2B cells for 7 days. For the in vivo experiments, 1, 5, or 10 mg/kg of sodium cyanide was orally administered to 4-week-old rats, only once, and the rats were sacrificed 1 or 7 days later. The total mRNA of the lung tissues was extracted and used for gene expression analysis.

**Figure 3:**
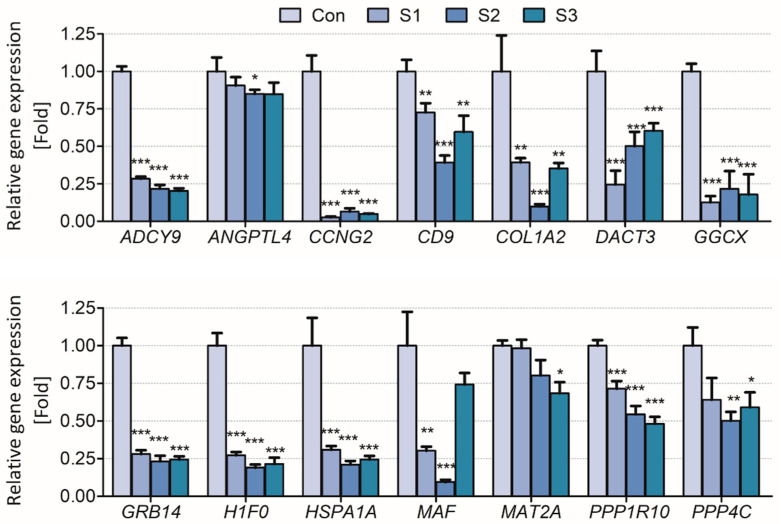
Validation of the candidate biomarkers of sodium thiocyanate exposure in BEAS-2B cells. Control (Con), vehicle; S1, 1 μM; S2, 10 μM; S3, 20 μM of sodium thiocyanate. Each result is the mean ± SEM of ≥three independent experiments. * *p* < 0.05, ** *p* < 0.01, *** *p* < 0.001.

**Figure 4:**
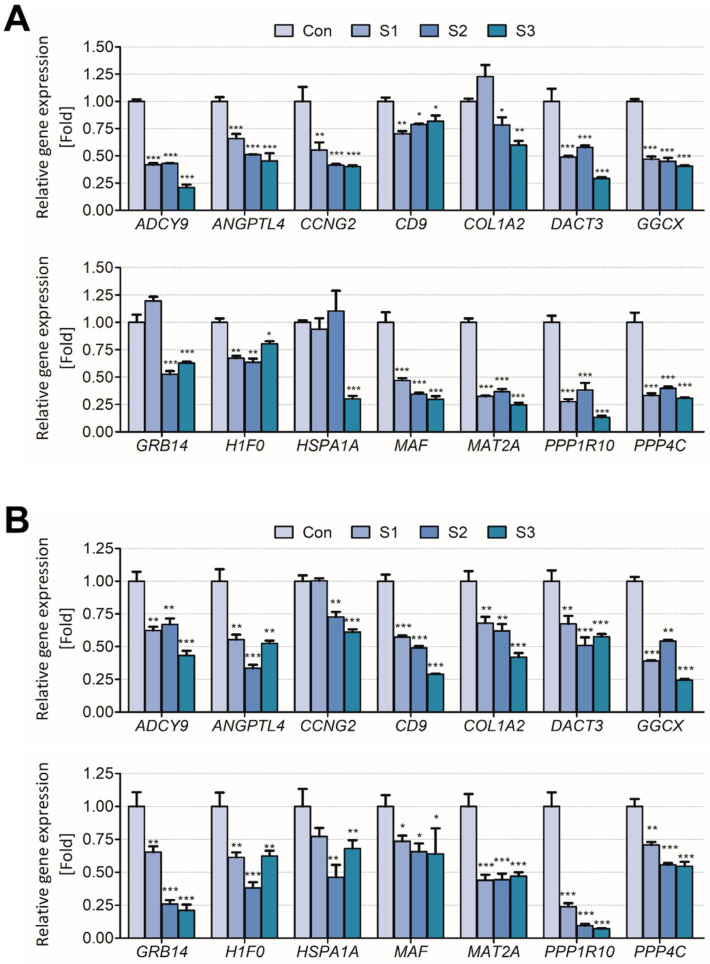
Validation of the candidate biomarkers of sodium cyanide exposure in 4-week-old rats. The mRNA levels of the candidates in the lungs of the rats were analyzed (**A**) 1 d or (**B**) 7 d after sodium cyanide exposure. Con, vehicle control; T1, 500 mg/kg; T2, 1000 mg/kg; T3, 2000 mg/kg of sodium cyanide. Each result is the mean ± SEM of ≥3 independent experiments. * *p* < 0.05, ** *p* < 0.01, *** *p* < 0.001.

The results showed that the biomarker candidates were mostly downregulated in vitro and in vivo. Especially, *ADCY9*, *CCNG2*, *COL1A2*, *GGCX*, *GRB14*, *H1F0*, and *HSP1A1* were significantly downregulated (>2 folds) after sodium thiocyanate exposure in normal human lung cells. Additionally, *ANGPTL4*, *MAT2A*, *PPP1R10*, and *PPP4C* were downregulated in a dose-dependent manner (Figure 3). In young rats, most biomarker candidates decreased in a dose-dependent manner. After 1 day of sodium cyanide exposure, *COL1A2*, *GRB14*, and *HSPA1A* were not significantly modulated in low-dose-exposure rats; however, they were significantly decreased in the rats 7 days after sodium cyanide exposure (Figure 4).

### 3.3. Characterization of the Candidate Biomarkers via PPI Network and Functional Enrichment Analyses

**Figure 5:**
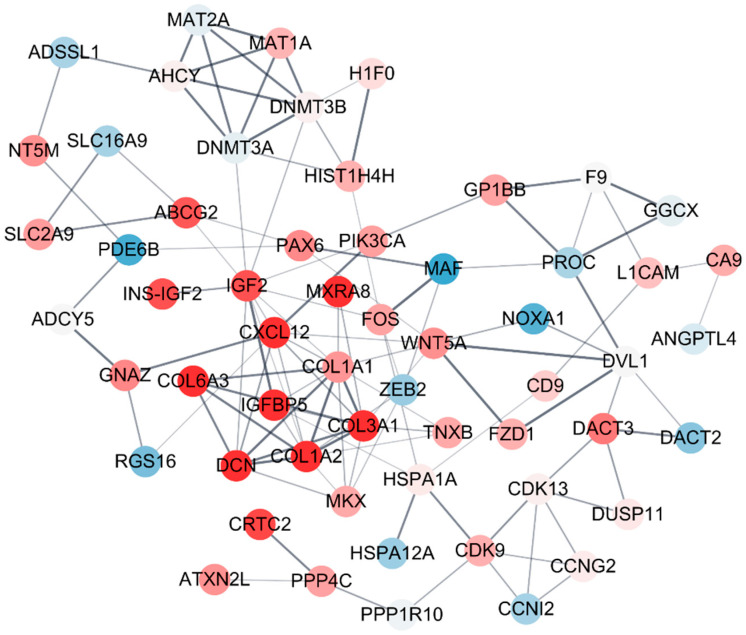
The protein–protein interaction (PPI) network of the biomarkers of sodium cyanide exposure. The differentially expressed genes (DEGs, ≥2 folds) were sorted and used for PPI analysis based on STRING DB. The module enrichment was based on the PPI network with a cutoff criterion of MCODE score >4. Each node and corner represent a gene and co-expression relationship, respectively. The pink and blue nodes indicate the upregulated and downregulated DEGs, respectively. The bold lines indicate that the connected nodes have a strong co-expression relationship.

PPI network provides an overview of the interaction web occurring inside a cell. The vast amount of sequencing data generated was utilized to better predict the functions of the proteins, as well as the interactions and functional associations among these proteins. Therefore, STRING and Cytoscape were used to build a protein interaction network of the molecular mechanisms (Figure 5). There were 58 nodes in the network of DEGs by sodium cyanide, in which *IGF*, *IGFBP2*, *ZEB*, and *WNT5A* were the central nodes, and if they are removed, the network crashes (Figure 5).

**Figure 6:**
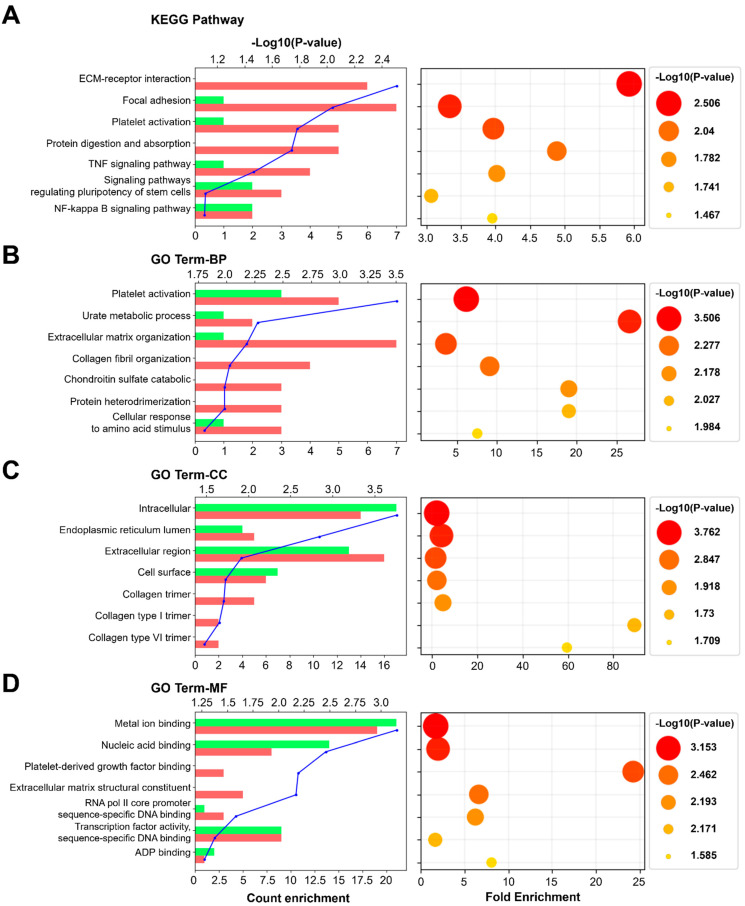
GO term and functional enrichment analyses of the biomarkers of sodium cyanide exposure. The top 7 most significantly affected pathways or GO terms are listed along the y-axes. The x-axes indicate count enrichment and fold enrichment. The pathways or terms with *p*-value < 0.05 were considered significantly enriched. The color and size of each dot indicate −Log_10_ (*p*-value). (**A**) KEGG pathway. (**B**) Biological process. (**C**) Cellular component. (**D**) Molecular function. GO, gene ontology.

GO term and functional enrichment analyses were performed using the 58 genes located on the nodes of the PPI network. Figure 6 shows the top seven most significantly affected GO terms or functions. The DEGs were annotated by the KEGG database and mapped to KEGG pathways. Among the GO terms, the common significant process was related to extracellular matrix regulation. The results were analyzed based on the KEGG database, and the expression of genes related to ECM receptor interaction, focal adhesion, platelet activation, protein digestion and absorption, TNF signaling pathway, signaling pathways regulating pluripotency of stem cell, and NFkB signaling pathway-related was significantly modulated. Similarly, the results of the GO term BP (biological process) showed that the expression of genes related to platelet activation, the urate metabolic process, extracellular matrix organization, collagen fibril organization, chondroitin sulfate catabolic, protein heterodimerization, and the cellular response to amino acid stimulus was significantly modulated. Additionally, the results of the GO term CC (cellular component) showed that the proteins encoded by the genes are localized to the intracellular region, endoplasmic reticulum lumen, extracellular region, or cell surface, or interact with collagen type 1 or type VI trimer. The results of the GO term MF showed that the gene products can bind to metal ions, nucleic acids, platelet-derived growth factor, structural constituents of the ECM, RNA pol II core promoter sequence-specific DNA, and ADP, and can act as transcription factors.

## 4. Discussion

Humans are exposed to hazardous substances through various routes, such as ingestion, inhalation, and touch, and these substances have various adverse effects on health [1]. Preventing exposure to hazardous chemicals and toxic substances is the fundamental way to protect workers; however, once they are exposed to hazardous substances, it is also important to measure the degree of exposure [3,4]. Exposure assessments of heavy metals are evaluated using actual chemical ions because these ions accumulate in the body and have a half-life of ≥10–30 years in the body [31]. However, most chemicals have short half-lives, ranging from under a few hours to days [32]. Thus, there are many cases where chemicals were not detected in human specimens for exposure assessment [13]. To overcome these limitations, omics approaches based on gene-based biomarker research are being actively conducted [33]. Here, a bioinformatics approach was used to identify biomarkers for sodium cyanide.

To identify reliable biomarkers of sodium cyanide exposure, the following two critical criteria were defined for candidate selection: (1) the mRNA level significantly changes in response to sodium thiocyanate treatment (FC > 2.0 or <0.50, adjusted *p*-value < 0.05); and (2) the mRNA level is significantly modulated by sodium cyanide exposure in both normal human lung cells and rat lungs.

To identify and validate biomarkers of sodium cyanide exposure, sodium thiocyanate was used for the in vitro experiments, whereas sodium cyanide was used for the in vivo experiments. Sodium cyanide, a highly poisonous inorganic compound, causes toxicity by inhibiting cytochrome C oxidase, thereby causing cellular hypoxia and cytotoxic anoxia and eventually leading to death [28]. However, as cyanide has a short half-life (t_1/2_ = 0.34–1.28 h), it is difficult to detect cyanide exposure via direct CN analysis after a long time [34]. Thiocyanate, the major metabolite of cyanide, has a longer half-life than that of cyanide (t_1/2_ = 96–192 h) [35]. Thiocyanate is approximately 7 times less toxic than cyanide; however, increased levels of thiocyanate in the body can adversely affect the thyroid and kidneys [36,37]. Therefore, sodium thiocyanate was directly administered to normal human lung cells, and sodium cyanide was orally administered to rats.

Various concentrations of sodium thiocyanate were administered to BEAS-2B cells for 4 h or 24 h. The 24 h treatment showed significant toxicity in a dose-dependent manner (Figure 2), whereas the 4 h treatment did not show strong toxicity (Figure 3). These results presumably reflect the fact that thiocyanate is a detoxified form of cyanide, which is produced during the detoxification mechanism in the body [38]. Hence, the three-point concentrations (1, 10, and 20 μM) were selected and applied to the subsequent experiments as low, medium, and high concentrations, respectively.

For the selection of biomarker candidates, the sodium thiocyanate was treated into BEAS-2B, and total mRNA was extracted and used for mRNA Quan-seq. The results showed that, among 25,737 mRNAs, 309 genes were significantly altered by more than 2 folds in the sodium thiocyanate treatment (Figure 2C). Additionally, the gene expression levels were mostly downregulated rather than upregulated, suggesting that sodium thiocyanate tends to suppress, rather than induce, the gene expression levels. To identify the significant biomarker candidate among DEGs, 309 DEGs were analyzed to distinguish the groups based on the biological processes in which they participated. The results showed that the DEGs by sodium thiocyanate related to the extracellular matrix, aging, and cell migration were more regulated than other GO terms were (Figure 2D,E). Clustering is a common technique used to categorize many experimental observations into groups or clusters. Hierarchical clustering is a widely applicable technique used to group genes or samples [39]. The clustering heatmap provides an interactive visualization of the classification of the DEGs upon sodium thiocyanate exposure. All the biomarker candidates were clustered into different subgroups on the columns according to expression patterns in the center grids of the heatmap (Figure 2F). Based on the GO results and significantly different expression levels, 14 biomarker candidates that were significantly up/downregulated were selected (Table 1).

To validate the applicability of the biomarker candidates for exposure assessment of sodium cyanide, their expression levels were analyzed in vitro (Figure 3) and in vivo (Figure 4). They were significantly downregulated both in vitro and in vivo even 7 d after the exposure, indicating that they can be used to evaluate sodium cyanide exposure even after some period from sodium cyanide exposure.

Characterization of the biological pathways of related genes provides important information to understand the molecular mechanisms underlying the effect of hazardous substances [40]. Especially, protein–protein interaction (PPI) networks provide many new insights into protein function and an overview of the web of interactions occurring in the cell [41]. Therefore, to characterize the biomarker candidates, STRING and Cytoscape were used to build the protein interaction network of the molecular mechanism among biomarkers. Biomarker candidates (Table 1) and their direct related genes were sorted and used for PPI analysis based on STRING DB. An enriched module is based on the PPI network with a cutoff criterion of MCODE score > 4. A connected node indicates that the two proteins that jointly contribute to a specific cellular process are considered functionally associated [42]. The results showed that all the biomarker candidates are organically related. Additionally, *IGF*, *IGFBP2*, *ZEB*, and *WNT5A* were located at the center of the network, indicating that they are key factors connecting the network of the biomarkers.

Functional enrichment analysis is used to determine classes of genes, providing a functional profile of that gene set and a better understanding of the underlying biological process [43]. Therefore, GO term and functional enrichment analyses were performed using the 58 genes located on the nodes of the PPI network. Figure 6 shows the top seven most significant GO terms or functions. Based on the total background in each pathway, we found the significantly enriched KEGG pathways for the DEGs. Among the GO terms, the common significant process is related to extracellular matrix regulation. The results showed that the expression of the genes related to “ECM receptor interaction” and “extracellular matrix organization” is significantly modulated. The GO term CC (cellular component) refers to the localization of a gene product. The significant GO term CC of our results includes the extracellular region, cell surface, collagen trimer, collagen type I trimer, and collagen type VI trimer, indicating that the protein products of the biomarker genes are located in the extracellular matrix and affect the regulation of the extracellular matrix. The GO term MF (molecular function) terms describe molecular-level activities performed by gene products. Our results showed that the gene products of the biomarkers are related to the structural constituents of the ECM. Thus, the biomarkers may contribute to the regulation or degradation of the extracellular matrix, which is essential to provide structural and biochemical support to the surrounding lung cells.

In conclusion, we identified the *ADCY5*, *ANGPTL4*, *CCNG2*, *CD9*, *COL1A2*, *DACT3*, *GGCX*, *GRB14*, *H1F0*, *HSPA1A*, *MAF*, *MAT2A*, *PPP1R10*, and *PPP4C* mRNAs as biomarkers of sodium cyanide exposure. All these biomarkers were significantly downregulated in vitro and in vivo. We functionally characterized them and established the impact of sodium cyanide on the transcriptomic profiles via in silico approaches. Our results suggest that the identified genes of biomarkers contribute to the regulation and degradation of the extracellular matrix, thereby eliciting a negative effect on the surrounding lung cells. However, the limitation of our study is that the significant modulation of exposure biomarkers was validated in lungs from rats. To provide direct evidence for the applicability of exposure biomarkers, we need to further validate whether these biomarkers significantly change in the body fluid from animal and clinical samples.

## Figures and Tables

**Table 1:** Biomarker candidates for assessment of sodium cyanide exposure.

Gene Symbol	Annotation			
	Description	Chromosome	Strand	Transcript_ID
*ADCY5*	Adenylate cyclase 5	Chr3	–	NM_183357
*ANGPTL4*	Angiopoietin like 4	Chr19	+	NR_104213
*CCNG2*	Cyclin G2	Chr4	+	NM_004354
*CD9*	CD9 molecule	Chr12	+	NM_001769
*COL1A2*	Collagen type I alpha 2	Chr7	+	NM_000089
*DACT3*	Dishevelled-binding antagonist of beta-catenin 3	Chr19	–	NM_001301046
*GGCX*	Gamma-glutamyl carboxylase	Chr2	–	NM_001142269
*GRB14*	Growth factor receptor bound protein 14	Chr2	–	NM_001303422
*H1F0*	H1 histone family member 0	Chr22	+	NM_005318
*HSPA1A*	Heat shock protein family A (Hsp70) member 1A	Chr6	+	NM_005345_3
*MAF*	v-maf avian musculoaponeurotic fibrosarcoma oncogene homolog	Chr16	–	NM_005360
*MAT2A*	Methionine adenosyltransferase 2A	Chr2	+	NM_005911
*PPP1R10*	Protein phosphatase 1 regulatory subunit 10	Chr6	–	NR_072994
*PPP4C*	Protein phosphatase 4 catalytic subunit	Chr16	+	NM_001303506

## Data Availability

Not applicable.
